# Molecular keypad controlled circuit for Ce(iii) and NO_3_^−^ ions recognition by μw synthesized silicon-embedded organic luminescent sensor†

**DOI:** 10.1039/c8ra07294a

**Published:** 2018-10-29

**Authors:** Navpreet Kaur, Gurjaspreet Singh, Jasbhinder Singh, Akshpreet Singh, Pinky Satija, Gurpreet Kaur, Jandeep Singh

**Affiliations:** Department of Chemistry, Lovely Professional University Phagwara–144411 Punjab India singhjandeep@gmail.com +91 9815967272; Department of Chemistry and Centre of Advanced Studies in Chemistry, Panjab University Chandigarh–160014 India; Department of Chemistry, Gujranwala Guru Nanak Khalsa College Civil Lines Ludhiana–141001 India

## Abstract

This report demonstrates the mimicking of an electronic circuit diagram towards Ce(iii) ion sensing response supported by molecular keypads. The probe naphthyl based triazole linked silatrane (NTS) was efficiently synthesized using a series of microwave mediated reactions. The luminescent sensor NTS was explored for the ion sensing response towards Ce(iii) ions using DMSO and DMSO : H_2_O 4 : 1 (v/v) as solvent media, respectively. The role of water in Ce(iii) ion sensing was detected as ‘turn-off’ response that contradicts the ‘turn-on’ with DMSO. Further, the sensing of NO_3_^−^ ions by NTS–Ce(iii) ensemble was associated with blue shift on absorption maxima. These mimicking response studies were sketched as circuit diagrams assisted by molecular keypad behaviour as IMPLICATION output logic gate.

## Abstract 2

This report demonstrates the mimicking of an electronic circuit diagram towards Ce(iii) ion sensing response supported by molecular keypads.

## Graphical abstract

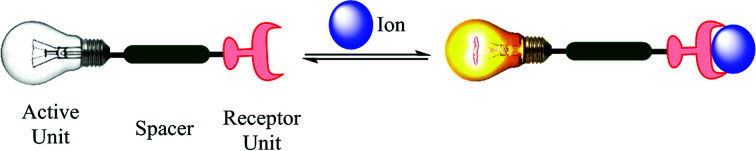

## Introduction

**Fig. 1:**
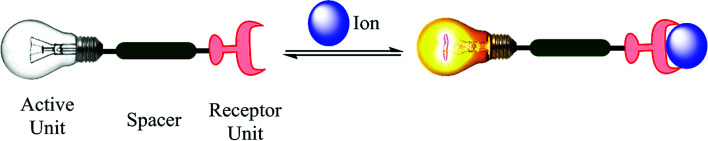
Representation of a fluorescent ion luminescent sensor.

Ion recognition studies work upon the principle of fluctuation in absorption or emission maxima upon interactions of an ion(s) with an ionophore. The role of chemical sensing is the exploration of its applications in different disciplines of chemistry and are not limited to, clinical biology and environmental science. Among the numerous kinds of chemical sensors, luminescence-based chemosensors display a set of advantages, since they have high sensitivity (single ion detection), versatility (bio-compatibility), low cost, easy application and good resolution, which are typical of photoluminescence spectroscopy.^1^ A chemosensor has two basic units (a) the receptor unit for selective binding and (b) the signal unit for reporting output through change in the optical properties (Fig. 1). The detection of target analyte is achieved by the molecular recognition followed by signal transduction.^2^ Among all type of compounds used as fluorescent chemosensors, organic luminophores are most widely studied due to their rich chemical structures, easy chemical modification and highly fluorescent quantum yield.^3,4^ The creation of luminophores through clean reaction methodologies have generated immense scientific interest in exploring copper(i)-catalyzed alkyne-azide cycloaddition (CuAAC).

There have been numerous citations pertaining to the transition metal ion sensing by using 1,2,3-triazoles but this is the first report (to the best of our knowledge) citing the use of ‘Click Silylation’ product for Ce(iii) ion analysis.^5–7^ ‘Click Silylation’ technique introduced a terminal siloxy group into the luminiphore bridged by 1,2,3-triazole linker.^8–15^ The naphthyl moiety (acting as active unit) linked *via* 1,2,3-triazole (spacer) has been explored as an ionic fluorophore can find potential application in bio-imaging technique owing to its specific excitation and emission UV values, unaltered by any interference from other metal ions.^7,16,17^ The triazole linker creates an effective link for host–guest binding yielding fluorescence quenching or enhancement, accompanied by batho- or hypso- chromic shift.^5^

Though cerium is classified as rare earth metal, it is still abundant in crust (66 μg g^−1^) and has been put into use in metallurgy, vehicle catalytic converters, ceramic materials and as heterogenous catalyst. In contrary, Ce(iii) ions hampers biological functioning by altering the immune systems and functioning of many essential body organs. The prolonged exposure to the moist areas rich in Ce(iii) ions can cause lung embolisms and liver damage.^18–21^ Thus, there is an urgent need of selective sensor for analysis and recovery of Ce(iii) ions in water, soil and industrial samples.^22^ The developments in cation sensing is interlinked to anion sensing and are driven by their major role in biology, industry and environment pollution. Anion sensing works on the displacement module where an anion binds to receptor by replacing an indicator, causing perturbations in absorption spectral values.^23–25^ The Schiff base framework acts as the backbone to assists binding of anions that may result into the significant visual changes after complexation.^26–29^

The mimicking of sensing behaviour can be processed as ‘0’ and ‘1’ input values in case of logic gates that is instantly processed by specific output changes as signals of molecular gates.^15,30–33^ So, far no Ce(iii) ion-ensemble sensing nitrate ions have been reported to the best of our knowledge. Thus, we herein report a new silicon capped 1,2,3-triazole linked naphthyl luminescent sensor (NTS) with regulated ON–OFF–ON switch module towards Ce(iii) and NO_3_^−^ ions. This type of behaviour has been mapped with the working of molecular switches that can work with an appropriate combination of chemical inputs.

## Experiment

### Materials and methods

All the syntheses were carried out under inert dry nitrogen atmosphere using vacuum glass line. The organic solvents were dried according to standard procedures.^34^ 2-Hydroxy-1-naphthaldehyde (Aldrich), 4-chlorobenzenamine (Aldrich), bromotris (triphenylphosphine) copper(i) [CuBr(PPh_3_)_3_] (Aldrich), tetrahydrofuran (THF) (CDH), triethylamine (Et_3_N) (SDFCL), triethanolamine (CDH), KOH (CDH), toluene (SDFCL), DMF (FINAR) were used as received. 3-Azidopropyltriethoxysilane (AzPTES) was synthesized by known procedure from literature.^35,36^ Infrared spectrum was obtained neat on a Thermo Scientific Fischer spectrometer. CHN analysis was obtained on Perkin Elmer Model 2400 CHNS elemental analyzer and Thermo Scientific Flash 2000 organic elemental analyzer. Multinuclear NMR (^1^H, ^13^C) spectra were recorded on a Bruker advance II 400 and on a Jeol (AL 300 MHz) spectrometer using CDCl_3_ as internal reference and chemical shifts were reported relative to tetramethylsilane. Melting points were uncorrected and measured in a Mel Temp II device using sealed capillaries.

### Synthesis of luminescent sensor 5

**Scheme 1:**
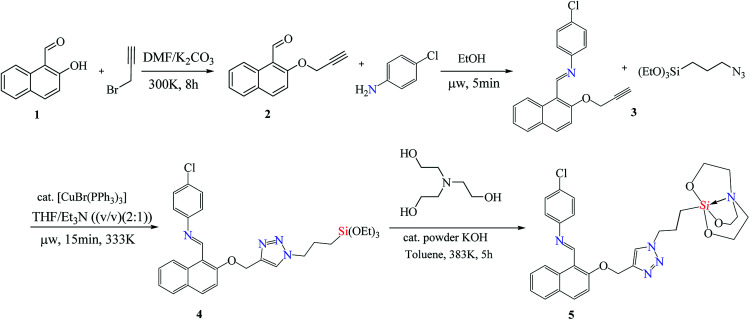
Reaction sequence followed for the synthesis of luminescent sensor NTS (5).

The entity 2 (Scheme 1) was synthesised from 2-hydroxy-1-naphthaldehyde 1 by a known procedure.^36–39^ The entity 3 was synthesized by microwave upon reacting equimolar quantity of 4-chloroaniline (0.18 g, 1.4 mmol) with 2 (0.3 g, 1.4 mmol) using 1 ml of ethanol as solvent at 60 °C. The yellow coloured solid product 3 was obtained within five minutes. The Schiff base alkyne 3 (0.38 g, 1.2 mmol) was treated with 3-azidopropyltriethoxysilane (0.29 g, 1.2 mmol) in equimolar ratio using THF : TEA 2 : 1 (v/v) (optimized as entry 2 in Table 1) as solvent and [CuBr(PPh_3_)_3_] as catalyst (0.01 mmol/alkyne function) in microwave at 60 °C for 15 minutes. The 1,4 triazole linked naphthyl terminated triethoxysilane 4 was extracted by solvent evaporation after catalyst filtration as stereospecific product.^40^ The silane 4 (0.63 g, 1.1 mmol) was processed by trans-esterification reaction with triethanolamine (0.18 g, 1.2 mmol) using toluene as solvent and anhydrous powdered KOH as catalyst to yield silatrane 5.

**Table 1:** Optimization of reaction conditions for μw assisted synthesis of triazole

Entry	Solvent ratio	Reaction duration (μw)	Yield^a^ (%)
1	THF : TEA 1 : 1 (v/v)	15 min	81
**2**	**THF : TEA 2 : 1 (v/v)**	**15 min**	**93**
3	THF : TEA 1 : 2 (v/v)	15 min	67
4	THF : TEA 3 : 1 (v/v)	15 min	74
5	THF : TEA 1 : 1 (v/v)	30 min	82
6	THF : TEA 2 : 1 (v/v)	30 min	93
7	THF : TEA 1 : 2 (v/v)	30 min	72
8	THF : TEA 3 : 1 (v/v)	30 min	79

a Determined by ^1^H NMR analysis of isolation of crude sample.

#### Synthesis of 3

Yield: 84%; mp: 121–122 °C; anal. calcd for C_20_H_14_ClNO (%): C, 75.12; H, 4.41; N, 4.38; found (%): C, 74.68; H, 4.51; N, 4.02. IR (neat, cm^−1^): 2920, 2943, 2986, 2828, 2041, 1474, 1397, 1113, 947, 787; ^1^H NMR (300 MHz, CDCl_3_) *δ* = 9.48 (d, *J* = 8.7 Hz, 1H), 9.15 (s, 1H), 7.87 (d, *J* = 9.1 Hz, 1H), 7.71 (d, *J* = 8.0 Hz, 1H), 7.51 (t, *J* = 7.3 Hz, 1H), 7.36 (d, *J* = 7.5 Hz, 1H), 7.31 (d, *J* = 4.8 Hz, 2H), 7.16 (t, *J* = 6.4 Hz, 2H), 4.83 (d, *J* = 2.3 Hz, 2H), 3.64 (s, 1H), 2.42 (t, *J* = 4.1 Hz, 1H).

#### Synthesis of 4

Yield 93%; viscous brown oil; anal. calcd for C_29_H_35_ClN_4_O_4_Si (%): C, 61.41; H, 6.22; N, 9.88; found (%): C, 61.38; H, 6.41; N, 9.25; IR (neat, cm^−1^): 2931, 2867, 2831, 1645, 1606, 1576, 1361, 1301, 1165, 1077, 1023, 887, 831, 797, 691; ^1^H NMR (300 MHz, CDCl_3_) *δ* = 9.45 (d, *J* = 8.7 Hz, 1H), 9.10 (s, 1H), 7.80 (d, *J* = 9.0 Hz, 1H), 7.64 (d, *J* = 8.0 Hz, 1H), 7.49–7.40 (m, 2H), 7.36–7.22 (m, 4H), 7.09 (d, *J* = 8.6 Hz, 2H), 5.32 (s, 2H), 4.21 (t, *J* = 7.1 Hz, 2H), 3.66 (q, *J* = 7.0 Hz, 6H), 1.95–1.81 (m, 2H), 1.09 (t, *J* = 7.0 Hz, 9H), 0.50–0.33 (m, 2H); ^13^C NMR (101 MHz, CDCl_3_) *δ* = 158.79, 158.43, 151.66, 134.04, 132.26, 131.94, 131.81, 131.02, 129.42, 129.14, 128.53, 128.47, 128.33, 125.91, 124.60, 122.35, 117.59, 114.20, 67.91, 63.52, 58.50, 57.98, 52.47, 25.59, 24.17, 18.42, 18.29, 7.40.

#### Synthesis of 5

Yield 87%; mp: 231–233 °C (decom.); anal. calcd for C_29_H_32_ClN_5_O_4_Si (%): C, 60.25; H, 5.58; N, 12.1; found (%): C, 59.78; H, 5.82; N, 12.27; IR (neat, cm^−1^): 2858, 1683, 1370, 1323, 1058, 977, 764, 577; ^1^H NMR (300 MHz, CDCl_3_) *δ* = 9.46 (d, *J* = 8.9 Hz, 1H), 9.16 (s, 1H), 7.86 (d, *J* = 9.0 Hz, 1H), 7.70 (d, *J* = 7.9 Hz, 2H), 7.60 (s, 1H), 7.52–7.39 (m, 4H), 7.13 (d, *J* = 8.4 Hz, 2H), 5.34 (s, 2H), 4.22 (t, *J* = 7.2 Hz, 2H), 3.60 (t, *J* = 5.7 Hz, 6H), 2.69 (t, *J* = 5.7 Hz, 6H), 1.99–1.71 (m, 2H), 0.33–0.17 (m, 2H); ^13^C NMR (101 MHz, CDCl_3_) *δ* = 158.94, 158.59, 151.77, 142.93, 134.08, 131.85, 130.98, 129.34, 129.13, 128.61, 128.54, 128.35, 125.89, 124.55, 122.93, 122.46, 122.37, 117.58, 114.27, 63.75, 57.49, 56.79, 53.46, 50.94, 26.31, 13.20; MS (ES^+^) calcd for [M + H]^+^ 578.19; found 578.18.

## Results and discussion

### Synthesis

The base catalysed nucleophilic substitution of 2-hydroxy-1-naphthaldehyde (1) with propargyl bromide resulted into an alkyne terminated product 2. This alkyne product was separated and was subjected to the microwave promoted condensation reaction with 4-chloroaniline yielding a Schiff base product 3 with terminal alkyne fragment. The Schiff base alkyne 3 with irradiated with microwaves in the presence of Cu(i) that catalysed cycloaddition reaction with 3-azidopropylsilane. The THF : TEA 2 : 1 (v/v) acted as solvent media and the reaction was complete within 15 minutes at the temperature of 60 °C. The resulting cyclo-addition product obtained contains 1,2,3-triazole linker with terminal ethoxysilyl group 4 in good yield. This product is hydrolytically unstable which restricts its reactivity and application in water-based systems. Thus, ethoxysilyl group was replaced by an atrane ring 5 by trans-esterification reaction, making it stable for use in various aqueous mediums. This luminescent sensor 5 was further used for chemo-sensing studies (labelled as NTS).

### UV-vis spectra

#### Cationic sensing

**Fig. 2:**
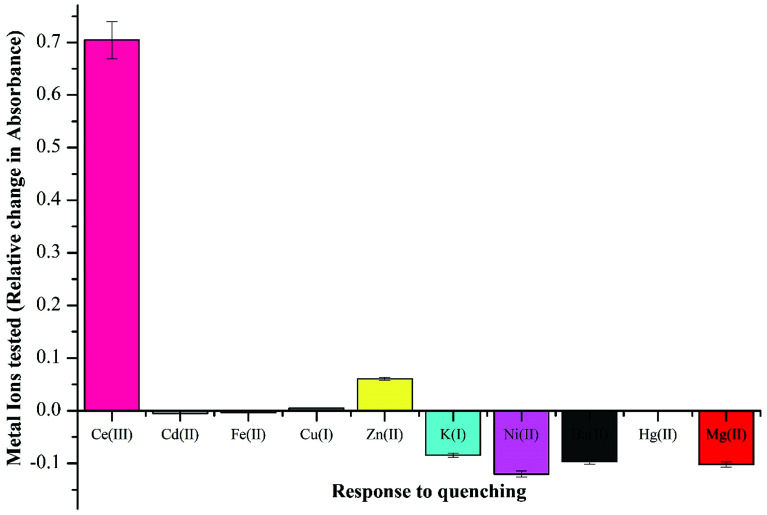
Competitive absorbance response recorded by luminescent sensor NTS with different cationic species using DMSO : H_2_O 4 : 1 (v/v) by addition of 5 equiv. of 20 μM metal ion solution with recorded change in maxima value at *λ* = 322 nm.

The absorbance spectral properties of NTS were examined solvatochromically using chloroform, dimethyl sulfoxide, dimethylformamide, and methanol, which demonstrated dimethyl sulfoxide to be the most suited solvent for photochromic analysis, owing to solubility and better detection studies. To record the metal-ion effect on absorption spectrum of NTS, 20 μM concentration of Ce(iii), Cd(ii), Fe(ii), Cu(i), Zn(ii), K(i), Ni(ii), Ba(ii), Hg(ii), Mg(ii) and Ag(i) ions were prepared and analysed in DMSO : H_2_O 4 : 1 (v/v). This demonstrated negligible quenching in absorption wavelength maxima, except for Ce(iii) ions, where a large stimulated change was recorded as plotted in Fig. 2. The activity of Ce(iii) ions in comparison to other metal ions owes to 6s^2^4f^1^5d^1^ electronic configuration wherein the high energy of 4f–5d configuration (6.3 eV) makes it more reactive. The quenching response of Ce(iii) ions is proposed to proceed through electron or energy transfer due to presence of unpaired electrons.^41,42^

**Fig. 3:**
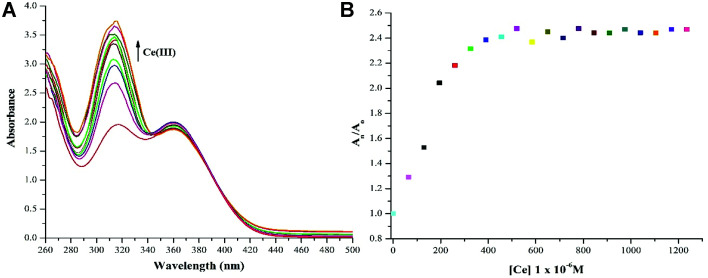
(a) ‘Turn-on’ response (*λ*_max_ = 322 nm) recorded with successive addition of 60 μM solution of Ce(iii) ions using DMSO as solvent owing to NTS–Ce(iii) ion interactions; (b) relative change in absorption maxima (*A*_n_/*A*_o_) with addition of 15 equiv. of 20 μM of Ce(iii) ions; (*A*_o_ = absorption maxima of NTS; *A*_n_ = absorption maxima with successive addition of Ce(iii) ions).

The ion sensing study was analysed in two different solvent media *i.e.* DMSO and DMSO : H_2_O 4 : 1 (v/v). The successive addition of 15 equivalent of 20 μM Ce(iii) in DMSO yielded a hyperchromic shift in the absorption maxima, commonly referred to as “turn-on” output optical response. The comparative “turn-on” behaviour among each peak decreased upon successive addition of Ce(iii) ions, which can be attributed to Internal Charge Transfer (ICT) interaction of NTS with Ce(iii) ions. The relative changes in absorption maxima with increasing Ce(iii) ion concentration have been shown in Fig. 3.

**Fig. 4:**
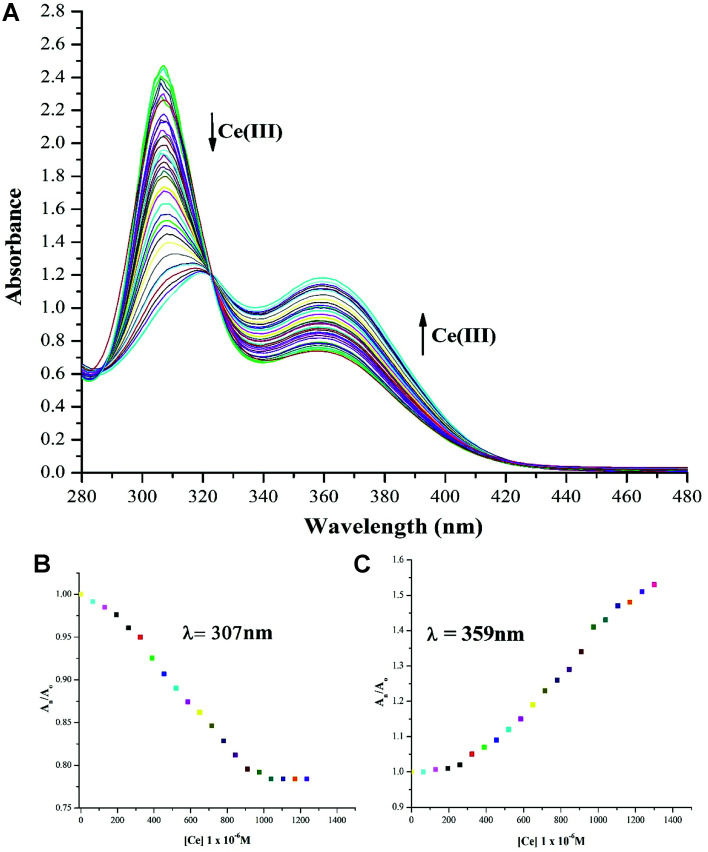
(a) ‘Turn-off’ response (*λ*_max_ = 307 nm) associated with red shift (to *λ*_max_ = 319 nm) recorded with successive addition of 20 μM solution of Ce(iii) ions using DMSO : H_2_O 4 : 1 (v/v) as solvent owing to NTS–Ce(iii) ion interaction which is associated with “turn-on” response (*λ*_max_ = 359 nm) creating an isosbestic point at 322 nm; (b) relative change in absorption maxima (*A*_n_/*A*_o_) with (*λ*_max_ = 307 nm) upon addition of 30 equiv. of 20 μM of Ce(iii) ions; (c) relative change in absorption maxima (*A*_n_/*A*_o_) with (*λ*_max_ = 359 nm) upon addition of 30 equiv. 20 μM of Ce(iii) ions; (*A*_o_ = absorption maxima of NTS; *A*_n_ = absorption maxima with successive addition of Ce(iii) ions).

To explore the utility of NTS in water based systems, the absorption maxima values were recorded and plotted for NTS with solvent mixture of DMSO : H_2_O 4 : 1 (v/v) as shown in Fig. 4. The studies performed with DMSO : H_2_O 4 : 1 (v/v) exhibit “turn-off” for absorption maxima at 319 nm accompanied by a blue shift to 307 nm. This titration process creates an isosbestic point at 322 nm accompanied by “turn-on” response for absorption maxima with *λ*_max_ of 359 nm, indicating the production of single component in response to NTS and Ce(iii) ion interactions. This observation can be attributed to the role of water interfering with NTS–Ce(iii)–DMSO interactions in the solvent mixture. The lowest detection limit of analysis of Ce(iii) ions using NTS probe is 60 μM.

#### Anionic sensing

**Fig. 5:**
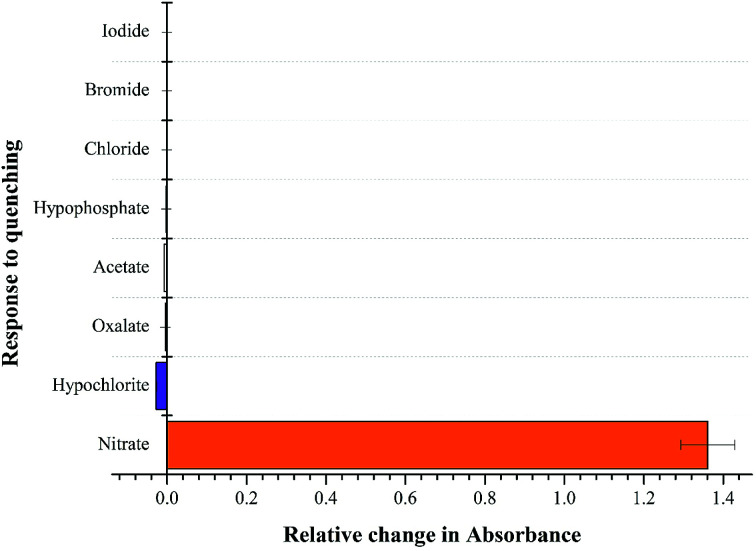
Competitive absorbance response recorded by excimer NTS–Ce(iii) complex with different anionic species using DMSO : H_2_O 4 : 1 (v/v) by addition of 5 equiv. of 13 μM anionic solutions with recorded change in maxima value at *λ* = 319 nm.

**Fig. 6:**
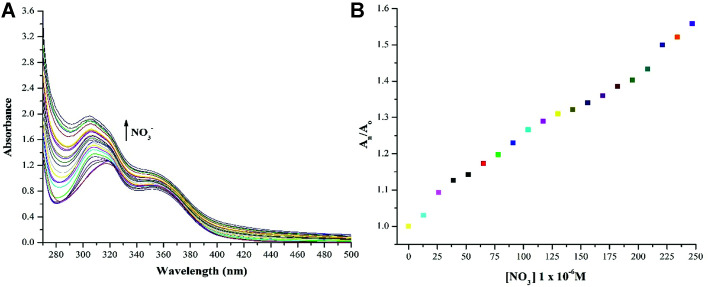
(a) “Turn-on” response (*λ*_max_ = 322 nm) associated with blue shift (to *λ*_max_ = 305 nm) was recorded with the successive addition of 10 μM solution of NO_3_^−^ ions using DMSO : H_2_O 4 : 1 (v/v) as solvent which is a result of [NTS–Ce(iii)] – NO_3_^−^ ion interactions; (b) relative change in absorption maxima (*A*_n_/*A*_o_) with addition of 13 μM of NO_3_^−^ ions; (*A*_o_ = absorption maxima of NTS–Ce(iii) ions; *A*_n_ = absorption maxima with successive addition of NO_3_^−^ ions).

**Fig. 7:**
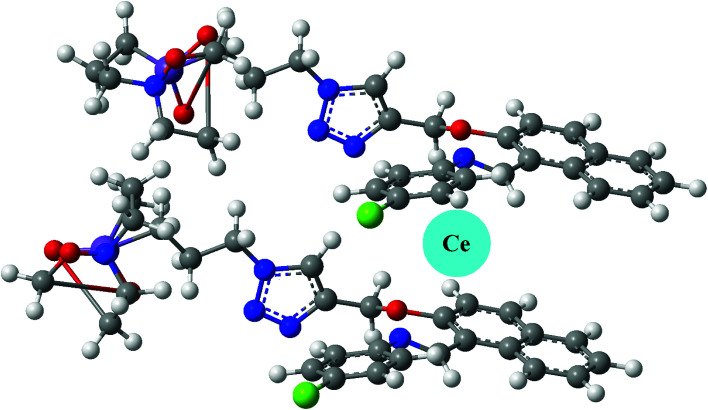
The binding mode of Ce^3+^ ions with NTS luminescent sensor.

NTS–Ce(iii) ensemble was tested for anion sensitivity using 13 μM solutions of different anions: NO_3_^−^, OCl^−^, C_2_O_4_^2−^, CH_3_COO^−^, Cl^−^, Br^−^, I^−^ in DMSO : H_2_O 4 : 1 (v/v) as shown in Fig. 5. The change in absorbance was observed with equimolar additions of NO_3_^−^ ions whereas insignificant changes were observed with OCl^−^ ions and no change was recoded with rest of the anions. The high selectivity displayed by rise in absorption maxima corresponds to “turn-on” response module for NTS–Ce(iii)–NO_3_^−^ system. There was recovery of original absorption maxima upon addition of 5 equiv. (13 μM) solution of NO_3_^−^ ions solution (Fig. 6). This chemo-sensing of anions using metals have been credited to photo electron transfer (PET) from receptor to luminescent sensor. The metal ion pre-organizes the complex to have a geometry compatible with host–guest binding. The lanthanide ensembles provide a platform for effective anion sensing in water, since aqua molecules being present in vacant coordination site can be displaced by anions. Thus, leading to spectral variations in metal-ensemble-anion system.^23–25^ On this basis, an expected binding mode was sketched (Fig. 7) with NTS : Ce(iii) ion binding in 1 : 1 fashion.

### Expected binding mode

NTS have been used as cationic chemo-sensor for cerium ion recognition, followed by nitrate ion assimilation. In order to render insight into the structural features, the geometry of ligand was optimised at DFT/B3LYP/6-31G level of theory. The optimized geometry had all frequencies indicating that obtained structure is a global minimum. Since, the binding of cerium as Ce(iii) depends upon the size of supramolecular cavity, it must held together by two molecules in a layered structure as shown in Fig. 7. This is supported by a fact that Ce(iii) is a larger ion, it cannot fit into a smaller cavity created by a single NTS molecule.^7^ Thus, the expected binding stoichiometric ratio for NTS : Ce(iii) is 2 : 1.

### Molecular keypad mimicking

**Fig. 8:**
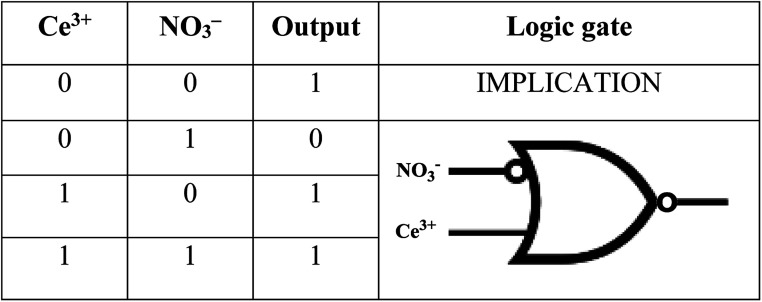
The relative absorbance intensity change observed with NTS upon addition of Ce^3+^ and NO_3_^−^ ions with ‘0’ and ‘1’ signifying ‘no change in *λ*_max_’ and ‘turn-off/turn-on’ response, respectively; along with the mimicking of response as IMPLICATION logic gate.

The plotting of ionic response as Boolean function by performing logical operation produces a response that can be implemented as an electronic switch control. These binding studies inspire us to design a [(receptor)–(Ce^3+^)–(NO_3_^−^)] binding model using molecular keypad. The simplification of input signal and output response as molecular keypad was designed as shown in Fig. 8. An alternate way of sensing behaviour for NTS was performed using NTS–NO_3_^−^ followed by successive addition of 20 μM of Ce(iii). The rise in signal intensity was observed with a pattern similar to that observed for NTS–Ce(iii) ensemble. These results evidently highlight the two-way sensing of both NO_3_^−^ ions.

### Circuit diagram

**Fig. 9:**
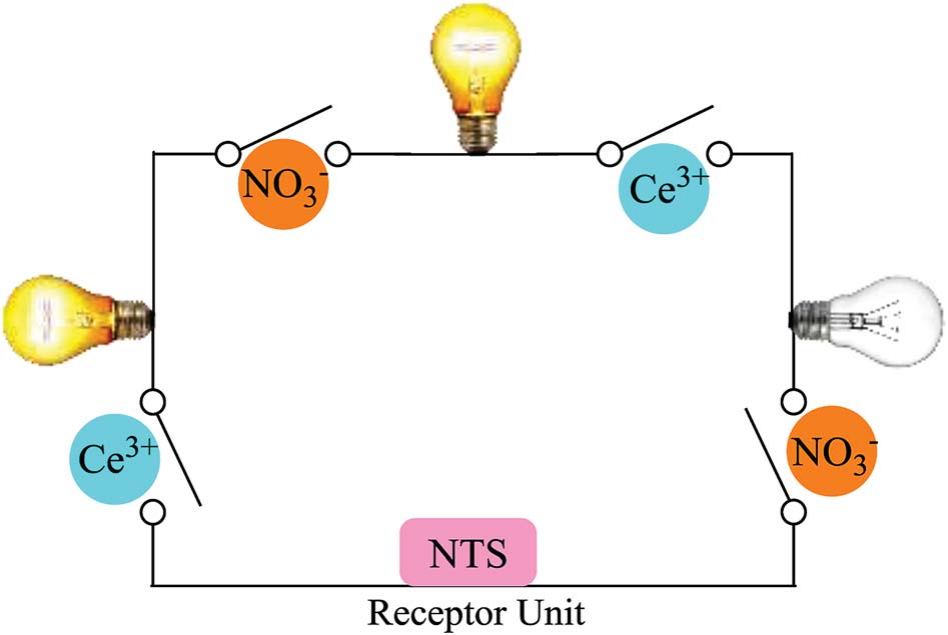
Ions controlled circuit diagram as a response with NTS.

The developments in electrical circuits to simplify the ion sensing behaviour will attract considerable scientific interest. The response obtained can be drafted as circuit board diagram (as shown in Fig. 9) with NTS as sensing unit and Ce(iii) and NO_3_^−^ ions as keys for circuit completion. The addition of Ce(iii) to NTS results into a change in absorption maxima whereas NO_3_^−^ ions do not alter the *λ*_max_ value. Further, the addition of NO_3_^−^ ions into NTS–Ce(iii) ensemble or Ce^3+^ ions to NTS–NO_3_^−^ ensemble depicts significant change in absorption maxima.

## Conclusion

The use of microwave radiations to create NTS luminescent sensor paved an effective pathway for clean, quick and efficient synthesis. The chemo-sensing of Ce(iii) ions in DMSO and DMSO : H_2_O 4 : 1 (v/v) solvent system resulted into the unique ‘turn-on’ and ‘turn-off’ responses, respectively. This ensemble was further utilised for NO^3−^ ion detection with a ‘turn-off’ response. The N, O, N cornered caged is generated that can efficiently trap Ce(iii) ions till the lower concentration limit of 60 μM. This behaviour of NTS–Ce(iii)–NO_3_^−^ system is mimicked as IMPLICATION logic gate that can be simplified as a circuit diagram.

## Conflicts of interest

There are no conflicts to declare.

## Supplementary Material

RA-008-C8RA07294A-s001
