# Supplementary material for: Molecular keypad controlled circuit for Ce(iii) and NO3− ions recognition by μw synthesized silicon-embedded organic luminescent sensor
Source: RSC Adv. 2018 Oct 29;8(64):36445–52. doi: 10.1039/c8ra07294a (PMC9088866; doi:10.1039/c8ra07294a)

Electronic Supplementary Material (ESI) for RSC Advances.
This journal is © The Royal Society of Chemistry 2018

Molecular keypad controlled circuit for Ce(III) and NO_3^- ions recognition by μw synthesized silicon–embedded organic luminescent sensor

Navpreet Kaur,[a] Gurjaspreet Singh,[b,*] Jasbhinder Singh,[a] Akshpreet Singh,[b] Pinky Satija,[b] Gurpreet Kaur,[c] Jandeep Singh,[a,*]

a, Department of Chemistry

Lovely Professional University, Phagwara–144411 (Punjab)

b, Department of Chemistry and Centre of Advanced studies in Chemistry

Panjab University, Chandigarh–160014

c Department of Chemistry

Gujranwala Guru Nanak Khalsa College, Civil lines, Ludhiana–141001

[*]Corresponding Author

Email: singhjandeep@gmail.com, Phone: +91 9815967272

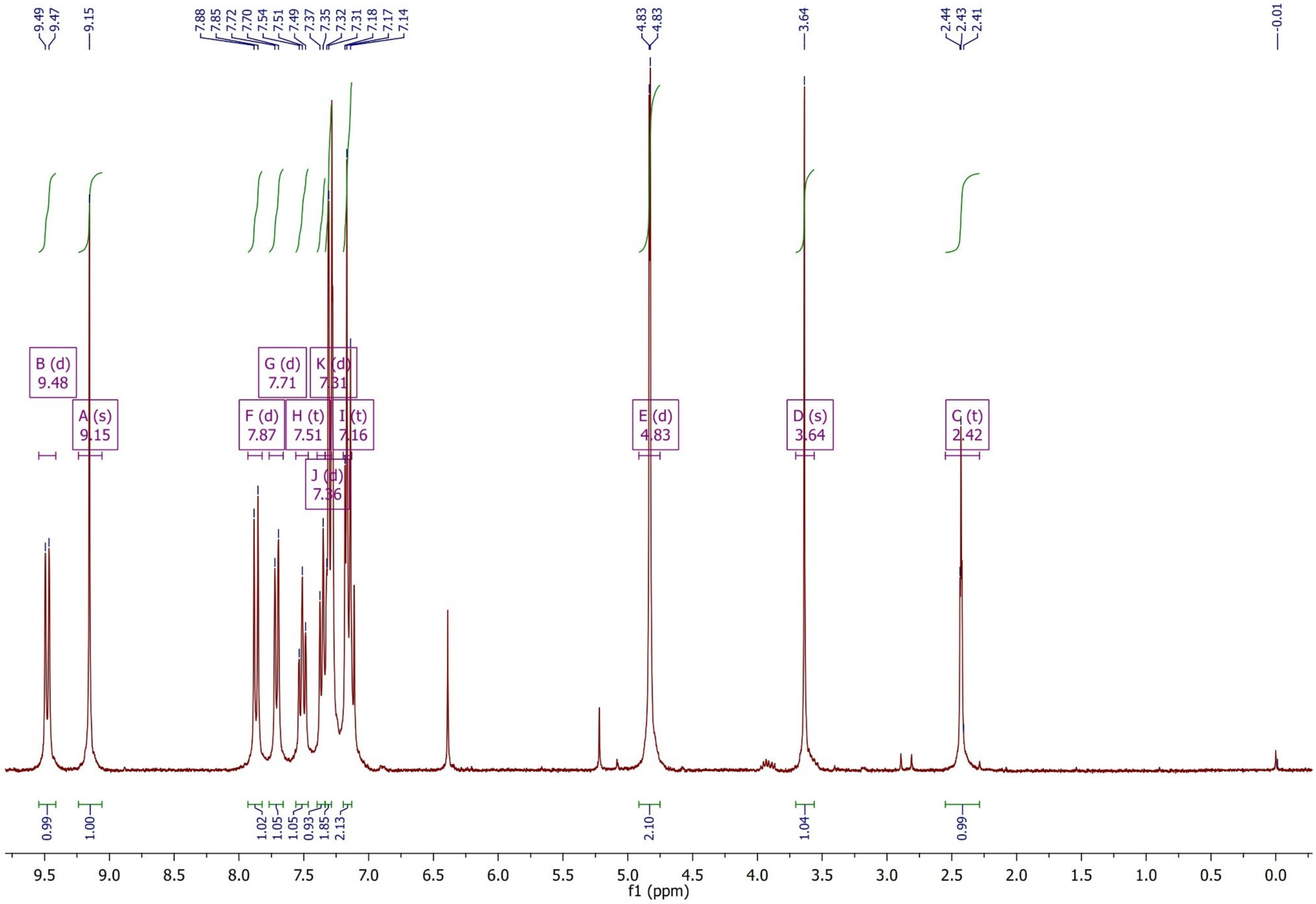

1H NMR of

compound 3

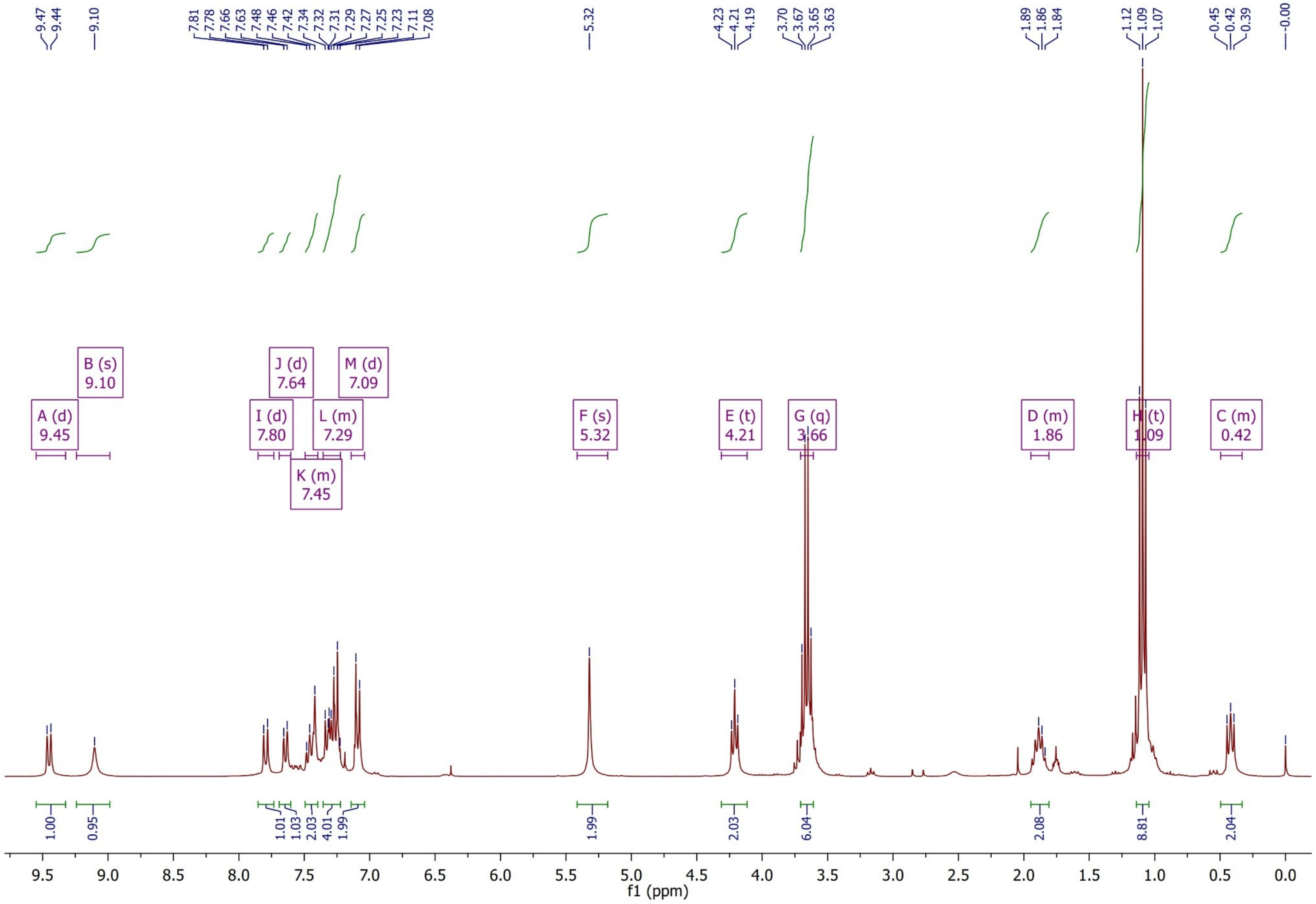

1H NMR of compound 4

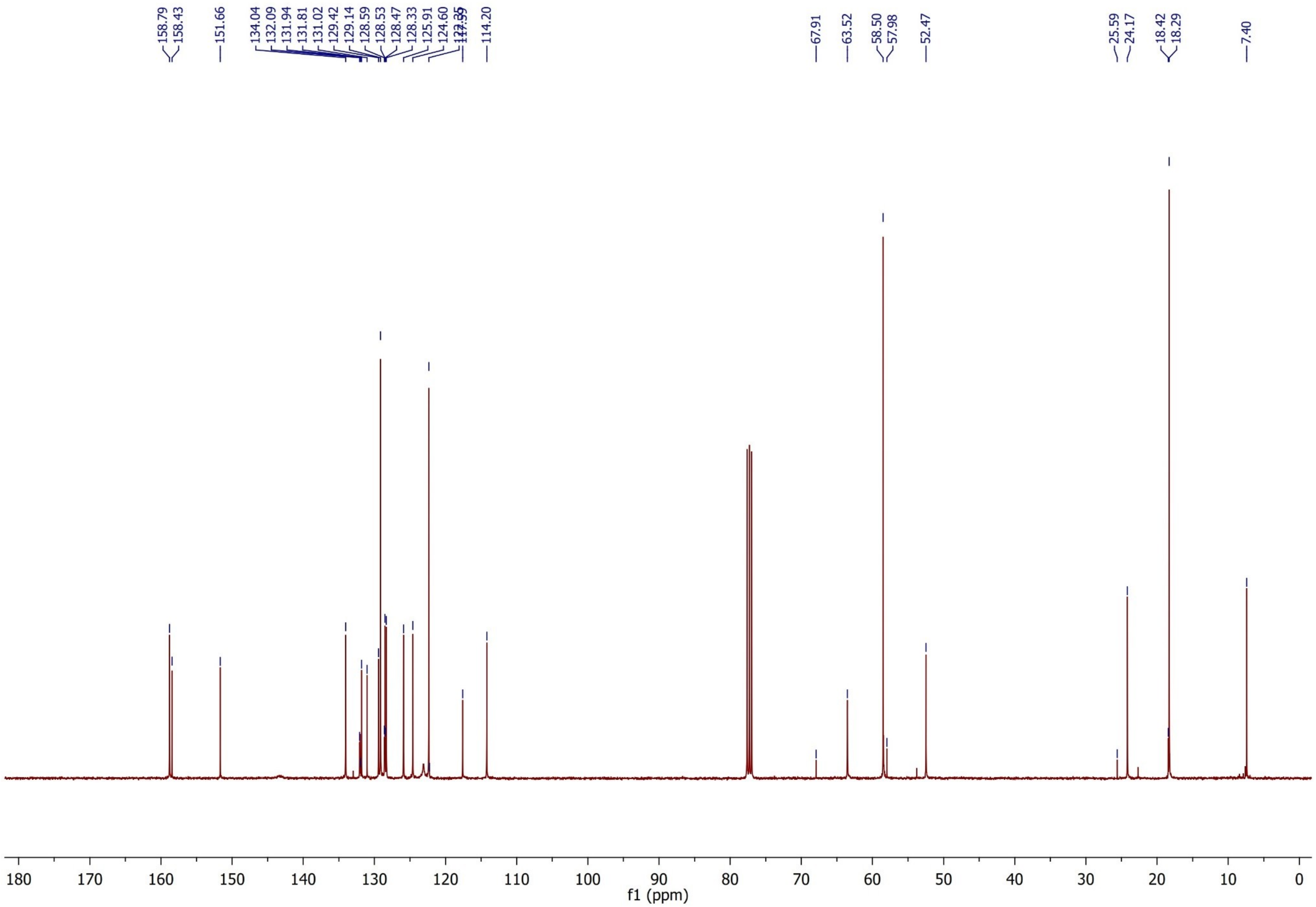

13C NMR of compound 4

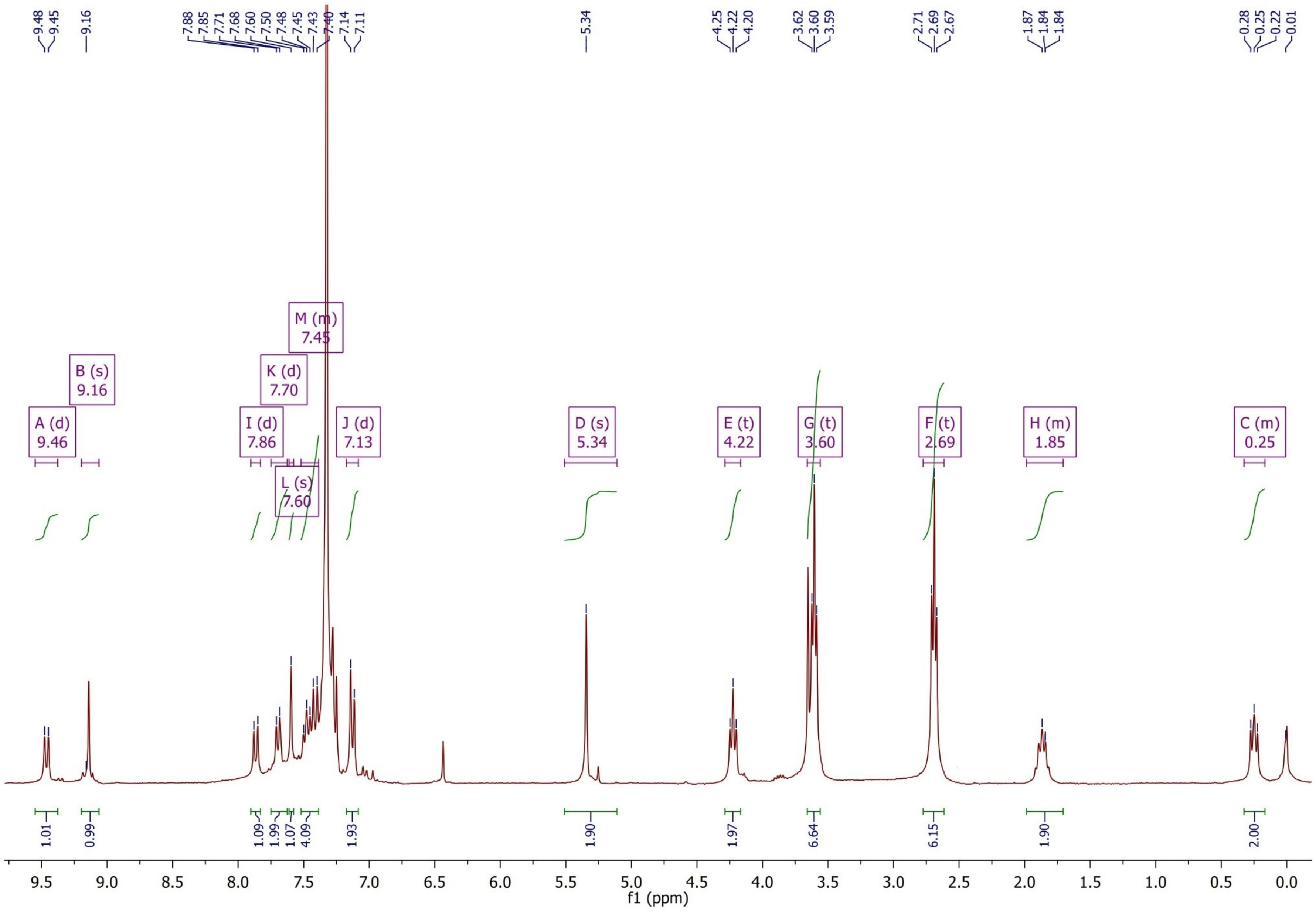

1H NMR of compound 5

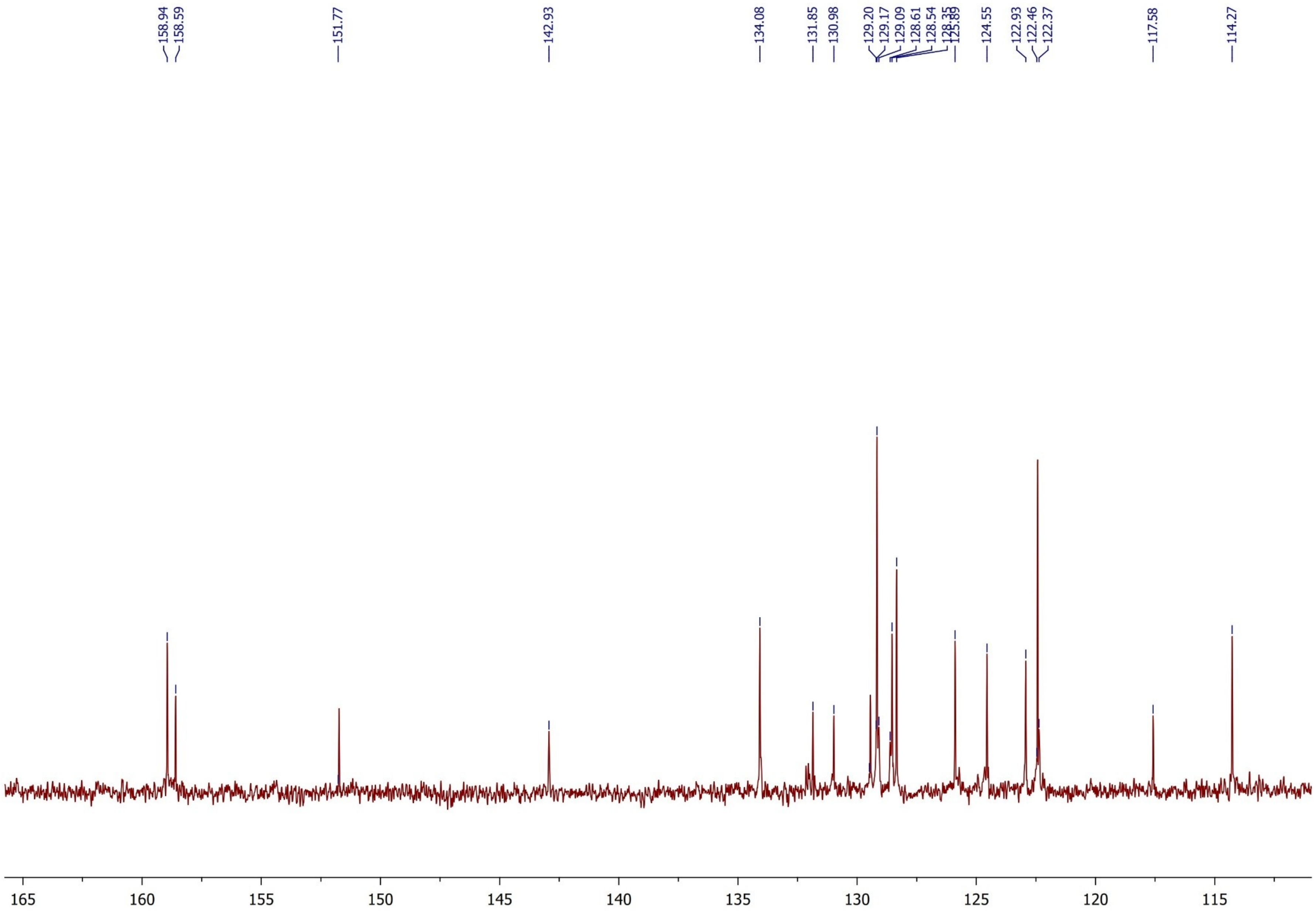

13C NMR of compound 5

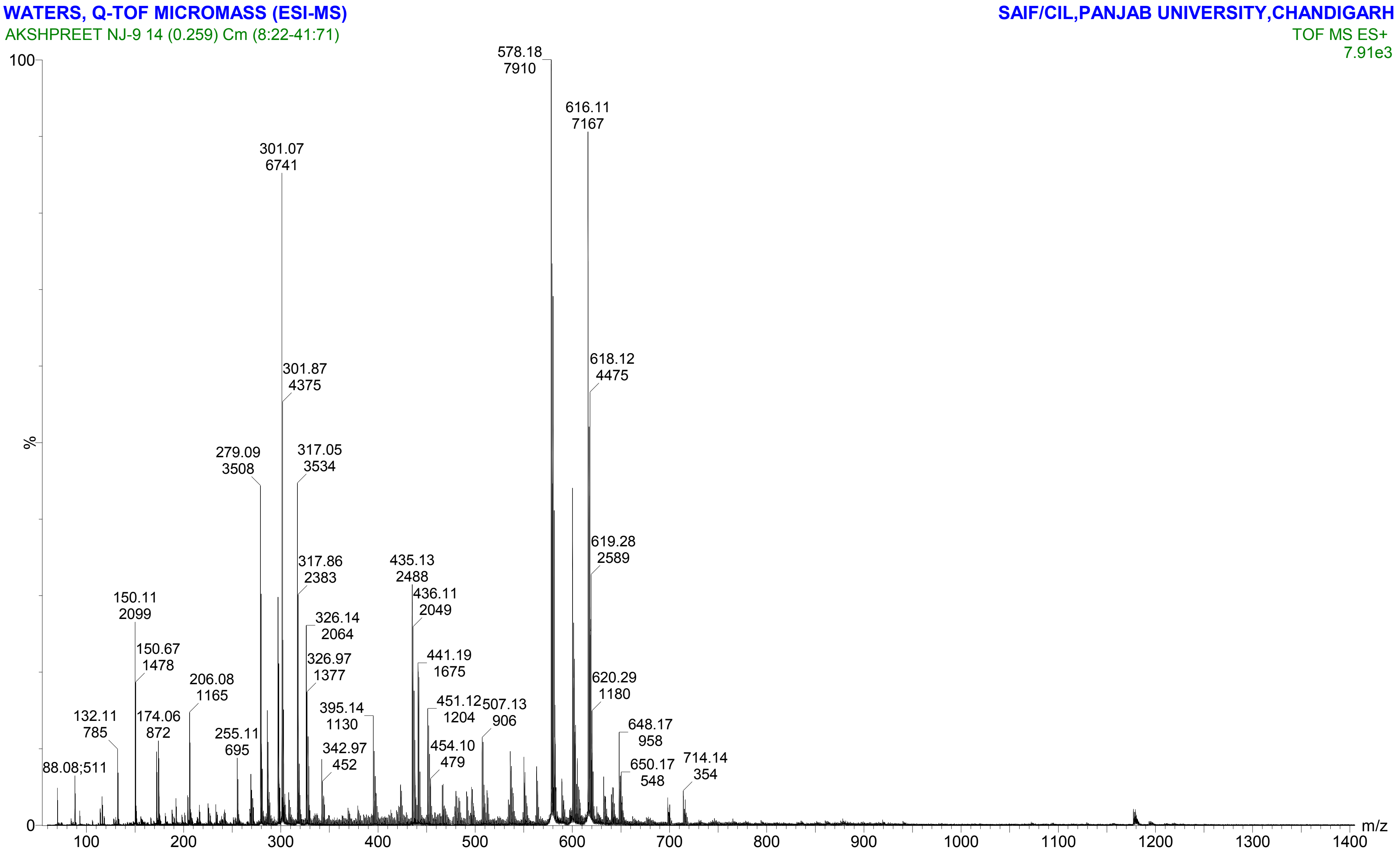

MS of compound 5

Cartesian coordinate

C	5.619984000	-1.746517000	-0.860862000
N	6.527714000	-1.161233000	0.127787000
C	5.865933000	-0.782164000	1.381335000
O	4.933051000	0.368801000	1.169200000
Si	4.905196000	0.788479000	-0.521719000
O	4.582073000	-0.749991000	-1.293673000
O	6.591377000	1.055742000	-0.886438000
C	7.394217000	-0.113447000	-0.421051000
H	8.082157000	0.234992000	0.350995000
H	7.943503000	-0.499299000	-1.280794000
H	5.280962000	-1.622823000	1.755806000
H	6.609958000	-0.467354000	2.113921000
H	6.183857000	-2.042687000	-1.746529000
H	5.091800000	-2.595536000	-0.429686000
C	3.779732000	2.204859000	-1.013182000
C	2.444464000	2.281602000	-0.239654000
H	3.597036000	2.103206000	-2.091562000
H	4.357200000	3.128610000	-0.877643000
C	1.599851000	3.442787000	-0.791290000
H	1.892522000	1.338946000	-0.336640000
H	2.629461000	2.433052000	0.829314000
H	2.115183000	4.396127000	-0.647954000
H	1.432438000	3.307409000	-1.867319000
N	0.304291000	3.604179000	-0.134389000
C	-0.767778000	2.757890000	-0.083304000
C	-1.744784000	3.475167000	0.581935000
H	-0.796409000	1.752065000	-0.469181000
N	0.002271000	4.813734000	0.485011000
N	-1.250309000	4.716407000	0.918586000
C	-3.152150000	3.080101000	0.864246000
H	-3.429804000	3.272575000	1.907398000
H	-3.844386000	3.628496000	0.212707000
C	-4.404154000	1.008459000	0.325549000
C	-4.337241000	-0.348511000	-0.037297000
C	-5.649918000	1.670209000	0.476821000
C	-5.572500000	-1.043311000	-0.325742000
C	-3.090511000	-1.111940000	-0.016904000
C	-6.826647000	0.998979000	0.235648000
H	-5.678219000	2.701955000	0.802000000
C	-6.828561000	-0.359098000	-0.178821000
C	-5.613359000	-2.392654000	-0.787540000
H	-3.222668000	-2.182464000	0.181175000
H	-7.776576000	1.510162000	0.360632000
C	-8.046741000	-1.041267000	-0.452406000

C	-6.812980000	-3.028556000	-1.047228000
H	-4.691482000	-2.930309000	-0.970633000
C	-8.046326000	-2.354642000	-0.872857000
H	-8.981047000	-0.501046000	-0.328544000
H	-6.808811000	-4.054932000	-1.400244000
H	-8.979049000	-2.867780000	-1.081194000
O	-3.213819000	1.649774000	0.576776000
N	-1.888745000	-0.647471000	-0.171413000
C	-0.774944000	-1.486619000	0.070506000
C	0.378771000	-1.307926000	-0.717153000
C	-0.748190000	-2.438501000	1.110635000
C	1.528407000	-2.071006000	-0.497606000
H	0.356728000	-0.569510000	-1.511136000
C	0.394990000	-3.208841000	1.340274000
H	-1.603975000	-2.546133000	1.767963000
C	1.508921000	-3.014922000	0.528804000
H	2.421806000	-1.905156000	-1.089197000
H	0.420960000	-3.932621000	2.145827000
Cl	3.007365000	-4.024903000	0.850675000

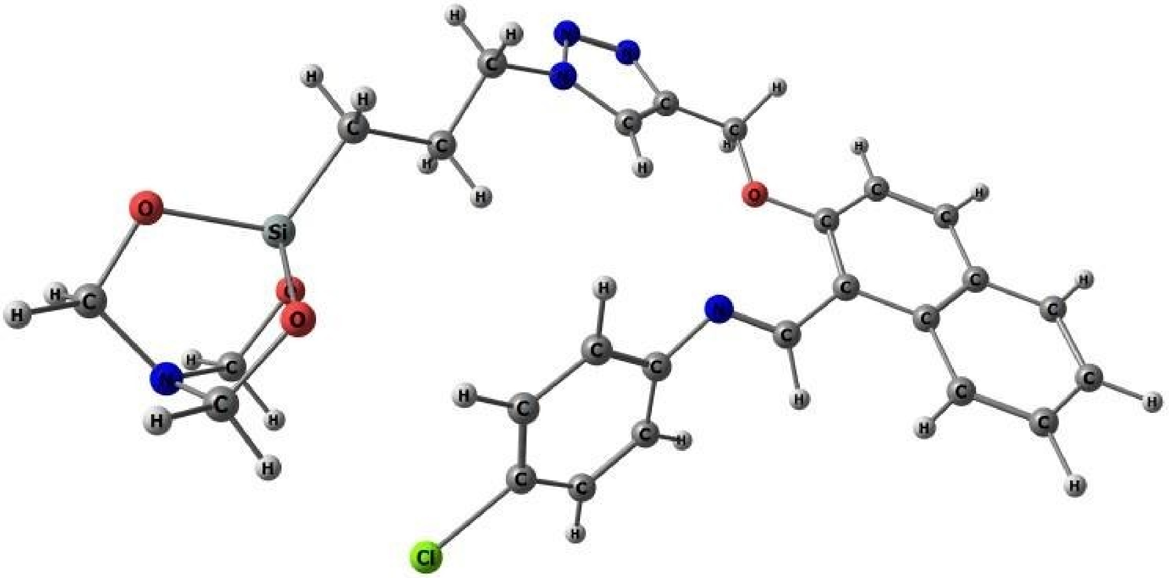

Supplement: RA-008-C8RA07294A-s001 [file RA-008-C8RA07294A-s001.pdf]
